# Network Pharmacology-Based Prediction of Mechanism of Shenzhuo Formula for Application to DKD

**DOI:** 10.1155/2021/6623010

**Published:** 2021-04-17

**Authors:** Xinmiao Wang, Haoyu Yang, Lili Zhang, Lin Han, Sha Di, Xiuxiu Wei, Haoran Wu, Haiyu Zhang, Linhua Zhao, Xiaolin Tong

**Affiliations:** ^1^Department of Endocrinology, Guang'anmen Hospital, China Academy of Chinese Medical Sciences, Beijing 100053, China; ^2^Graduate College, Beijing University of Traditional Chinese Medicine, Beijing 100029, China; ^3^Laboratory of Molecular and Biology, Guang'anmen Hospital, China Academy of Chinese Medical Sciences, Beijing 100053, China; ^4^Department of Endocrinology, Affiliated Hospital to Changchun University of Chinese Medicine, Changchun 130021, China

## Abstract

**Background:**

Shenzhuo formula (SZF) is a traditional Chinese medicine (TCM) prescription which has significant therapeutic effects on diabetic kidney disease (DKD). However, its mechanism remains unknown. Therefore, this study aimed to explore the underlying anti-DKD mechanism of SZF.

**Methods:**

The active ingredients and targets of SZF were obtained by searching TCMSP, TCMID, SwissTargetPrediction, HIT, and literature. The DKD target was identified from TTD, DrugBank, and DisGeNet. The potential targets were obtained and PPI network were built after mapping SZF targets and DKD targets. The key targets were screened out by network topology and the “SZF-key targets-DKD” network was constructed by Cytoscape. GO analysis and KEGG pathway enrichment analysis were performed by using DAVID, and the results were visualized by Omicshare Tools.

**Results:**

We obtained 182 potential targets and 30 key targets. Furthermore, a “SZF-key targets-DKD” network topological analysis showed that active ingredients like M51, M21, M5, M71, and M28 and targets like EGFR, MMP9, MAPK8, PIK3CA, and STAT3 might play important roles in the process of SZF treating in DKD. GO analysis results showed that targets were mainly involved in positive regulation of transcription from RNA polymerase II promoter, inflammatory response, lipopolysaccharide-mediated signaling pathway, and other biological processes. KEGG showed that DKD-related pathways like TNF signaling pathway and PI3K-Akt signaling pathway were at the top of the list.

**Conclusion:**

This research reveals the potential pharmacological targets of SZF in the treatment of DKD through network pharmacology and lays a foundation for further studies.

## 1. Introduction

Diabetic kidney disease (DKD) is one of the most common chronic microvascular complications of diabetes. It may be caused and shaped by the interaction of many factors such as endoplasmic reticulum dysfunction, high sugar-mediated generation of terminal advanced glycation endproducts (AGE), increased activation of the renin angiotensin aldosterone system, increased generation of reactive oxygen species (ROS), and activation of extracellular matrix (ECM) and protein kinase C [1, 2]. It is reported that the incidence of DKD is about 40% in the diabetic population [3]. Furthermore, with the increasing incidence of diabetes, the incidence of DKD is increasing yearly [4]. Therefore, it is important to intensify studies of the pathogenesis of DKD and the search for effective intervention targets.

Shenzhuo formula (SZF) as a traditional Chinese medicine (TCM) prescription has certain advantages in the treatment of DKD [5].It is created by Tong Xiaolin, an academician at the Chinese Academy of Sciences, and his team. This formula was based on the pathogenesis of qi deficiency blood stasis, and the classic prescription of Didang decoction. Years of clinical studies have shown that SZF can effectively increase the glomerular filtration rate, reduce 24-hour urinary protein and kidney damage, and reverse kidney disease when used early [5, 6]. However, due to the diversity of TCM compounds and complexity of *in vivo* processes, the systematic mechanism research of SZF has been hindered.

Recently, network pharmacology has been developed rapidly with the use of multiomics, high-throughput screening, network visualization and analysis, or other techniques [7–9]. It can help to reveal the network structure of drug action [10] and provide possibilities for exploring the mechanism of action of TCM compounds. Therefore, this study aimed to shed light on the underlying mechanisms of SZF in DKD treatment using a network pharmacology approach.

## 2. Methods

### 2.1. Research Tools

The Chinese Traditional Medicine System Pharmacological Database Analysis Platform (TCMSP, http://lsp.nwu.edu.cn/tcmsp.php) [11], Traditional Chinese Medicine Integrated Database (TCMID, http://www.megabionet.org/tcmid/) [12], SwissTargetPrediction (http://www.swisstargetprediction.ch/) [13], and HIT (http:lifecenter.biosino.org/hit/) [14] were used to access to SZF ingredients and targets. (2) The Therapeutic Target Database (TTD, http://bidd.nus.edu.sg/group/cjttd/) [15], DrugBank (https://www.drugbank.ca/) [16], and DisGeNet (http://www.disgenet.org/) [17] were used to get the targets' proteins of DKD. (3) The protein-protein interaction (PPI) network was obtained online using STRING (http://string-db.org) [18]. Compositional software Cytoscape 3.2.1 (http://www.cytoscape.org/) [19] was used to carry out network topology analysis and construct SZF-key targets-DKD network. The Database for Annotation, Visualization and Integrated Discovery (DAVID, http://david.ncifcrf.Gov) [20] was used for Gene Ontology (GO) and Kyoto Encyclopedia of Genes and Genomes (KEGG) analysis. The Omicshare Tools (https://www.Omicshare.com/) were used for visual analysis of GO and KEGG results.

### 2.2. Collection of Major Chemical Constituents

We relied on TCMSP, TCMID database, and literatures mining to search for the chemical constituents of SZF (Hedysarum Multijugum Maxim, Radix Salviae, Hirudo, and Radix Rhei Et Rhizome).

### 2.3. Screening of Active Compounds

As we all know, TCM drugs enter human body and then take effect through absorption, distribution, metabolism, and excretion (ADME) processes. Among them, oral bioavailability (OB) and drug similarity (DL), the key parameters of ADME components, were used as the screening criteria for active ingredients in this study. In this section, we used TCMSP to collect active compounds and their ADME properties. And then the active compounds that meet “OB ≥ 30%, DL ≥ 0.18” were selected as potential active ingredients.

### 2.4. Prediction of Targets

SwissTargetPrediction and HIT databases were used to collect the drug targets. In addition, TTD, DrugBank and DisGeNet databases were used to search for DKD targets by entering the key words of “diabetic kidney disease” and “diabetic nephropathy.” Further, we matched SZF targets with DKD targets to obtain common targets.

### 2.5. Network Construction and Analysis

PPI network of common targets was obtained using STRING. Furthermore, the PPI network topology analysis was carried out using Cytoscape 3.2.1 software and then key targets were obtained. To further explore the interactions between the active ingredients and their related targets at a system level, a “SZF-key targets-DKD” network was constructed by Cytoscape3.2.1.

### 2.6. GO and KEGG Analysis

GO analysis is widely used for gene function classification and mainly includes the molecular function (MF), biological processes (BP), and cellular components (CC) [21]. In this step, we used the DAVID tool for GO and KEGG pathway analysis. Then, we used Omicshare Tools for visual display.

## 3. Results

### 3.1. Screening of Candidate Components in SZF

Through TCMSP and TCMID database, a total of 87 active compounds of Hedysarum Multijugum Maxim, 210 of Radix Salviae, 35 of Hirudo, and 92 of Radix Rhei Et Rhizome were obtained. Then by ADME (OB ≥ 30%, DL ≥ 0.18) screening, a total of 101 active compounds were selected, including 20 active compounds of Hedysarum Multijugum Maxim, 65 of Radix Salviae, and 16 of Radix Rhei Et Rhizome (in this section, because Hirudo could not be found in TCMSP database, its ADME parameters could not be obtained and did not participate in screening). In addition, through literature mining, another 4 active compounds were collected, including 2 active compounds of Hedysarum Multijugum Maxim [22, 23], 1 of Radix Salviae [24], and 1 of Radix Rhei Et Rhizome [25].

### 3.2. Target Prediction

After matching SZF targets with DKD genes, a total of 182 common targets of SZF were obtained. We only show 50 of them in Table 1. And full information of 182 common targets is displayed in Table 2.

### 3.3. Construction and Analysis of Network Maps

**Figure 1:**
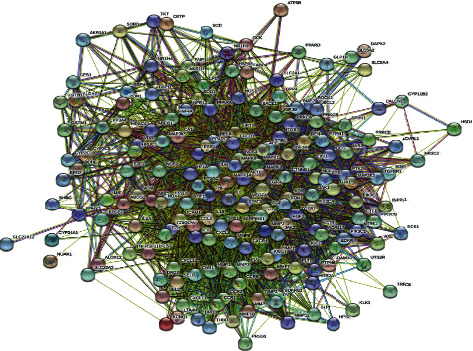
PPI network of the 182 common targets.

**Figure 2:**
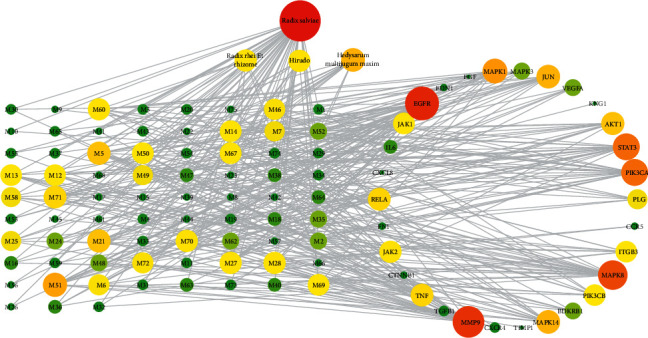
“SZF-key targets-DKD” network. The nodes were visualized with degree. The larger and the redder the node, the higher the degree it was. M1-75 stand for the active ingredients whose full names are shown in Table 4.

The PPI network of the 182 common targets was obtained using STRING (Figure 1). Then, we used Cytoscape 3.2.1 to obtain 30 key targets by network topology analysis with inclusion criteria of “degree ≥ 2 times of the median, closeness centrality ≥ median, betweenness centrality ≥ median” (Table 3). Next, we constructed a “SZF-key targets-DKD” network by Cytoscape3.2.1 (Figure 2).

### 3.4. GO and KEGG Analysis

**Figure 3:**
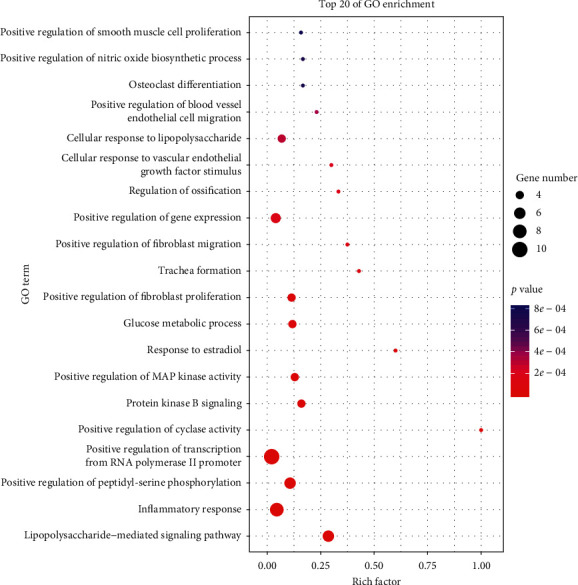
Top 20 enrichments in GO analysis.

The DAVID was used to carry out GO analysis. And the GO terms were visualized by the Omicshare Tools (Figure 3). The GO analysis results showed that targets were mainly involved in positive regulation of transcription from RNA polymerase II promoter, inflammatory response, lipopolysaccharide-mediated signaling pathway, positive regulation of peptidyl-serine phosphorylation, and other biological processes. As the top 20 GO enrichment items listed, DKD is relevant to kinds of BP in body abnormalities, and SZF is likely to regulate these items and then play an anti-DKD role.

**Figure 4:**
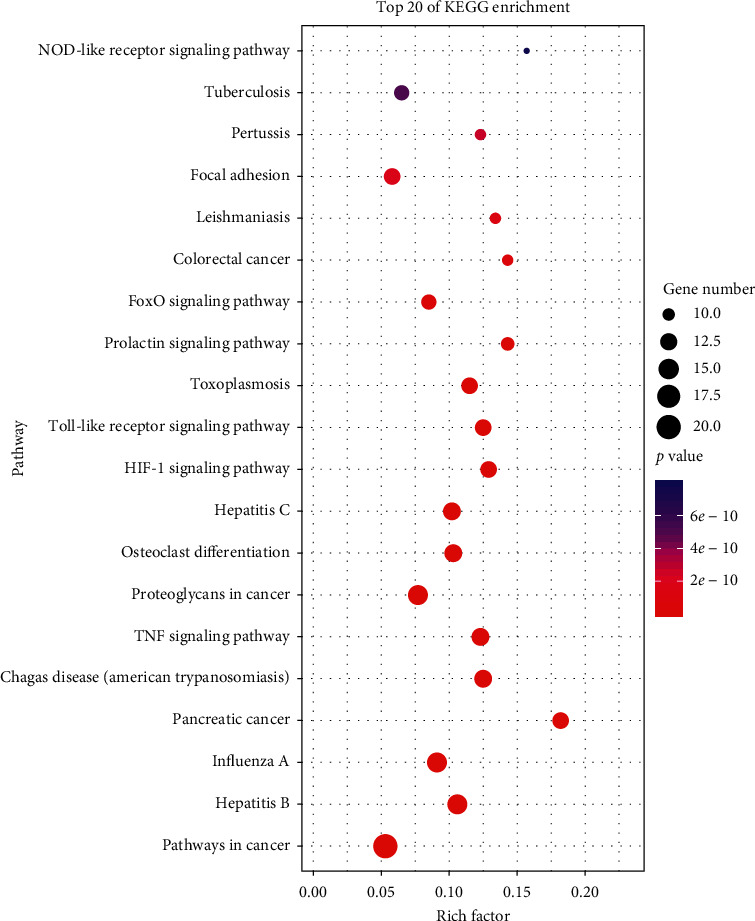
Top 20 KEGG pathway enrichments.

KEGG pathway enrichment analysis showed that a total of 104 pathways were obtained. The top 20 pathways are displayed in Figure 4, which include TNF signaling pathway, HIF-1 signaling pathway, Toll-like receptor signaling pathway, FoxO signaling pathway, NOD-like receptor signaling pathway, and so on.

## 4. Discussion

Previous studies have suggested that SZF has a therapeutic effect on DKD [5,6]. However, the potential mechanisms of SZF treating in DKD have not been fully explained. In this study, we mainly applied network pharmacology to explore it. Firstly, a total of 140 potential active compounds and 182 common targets of SZF and DKD were obtained after screening of active compounds and mapping of targets. Then, we constructed two networks, including the PPI network of 182 common targets and SZF-key targets-DKD network, and then applying GO and KEGG enrichment analysis to explore the regulation mechanism of SZF in treating DKD.

Through the SZF-key targets-DKD network, we could know that most active ingredients were linked with no less than one target, which indicated the character of multi-target of TCM active ingredients. In the meanwhile, different active compounds from different herbs acted on the same targets, which demonstrated that SZF had a synergistic effect in treating DKD. In addition, there were 8 active ingredients whose degrees were greater than 2 times of average in SZF-key targets-DKD network topology analysis. Interestingly, 3 of them had been proven to have kidney protection effect by experiments. For example, quercetin liposomes had renal protective effects of reducing oxidative stress, attenuating AGE expression, and delaying the progression of DKD [26]. Luteolin attenuated DKD mainly via suppression of inflammatory response and oxidative response [27]. Ursolic acid alleviated renal damage in type 2 diabetic db/db mice by downregulating proteins in the angiotensin II type 1 receptor-associated protein/angiotensin II type 1 receptor signaling pathway to inhibit extracellular matrix accumulation, renal inflammation, fibrosis, and oxidative stress [28]. These results were coincident with our predictions, which suggested that active ingredients with higher degree might play an important role in the treatment of DKD. Meanwhile, we discovered five active ingredients (M5, M27, M28, M60, and M70) that were likely to have renal protection effect but had not been verified up to now.

Moreover, the results of the SZF-key targets-DKD topology analysis also showed that there were 5 targets whose degrees were greater than 2 times of the average. Particularly, 3 of these had been proven to be closely related with DKD. For instance, EGFR activation had a significant role in activating pathways that mediate podocyte injury and loss in diabetic nephropathy [29]. Downregulated expression of MMP-9 could promote the process of DKD [30]. STAT3 inhibition could hinder the development and progression of DKD in diabetic patients [31].

As shown in GO analysis, the potential targets of SZF acting on DKD were mainly associated with various biological processes, such as lipopolysaccharide-mediated signaling pathway, inflammatory response, positive regulation of cyclase activity, protein kinase B signaling, positive regulation of MAP kinase activity, and response to estradiol, which had a strongly direct correlation with the pathogenesis of DKD [32–38].

Similarly, KEGG pathway enrichment analysis showed that SZF took an anti-DKD effect by multiple pathways. Through further research, we found that some pathways had been already verified to exert anti-DKD potential by experiments, such as TNF signaling pathway [39], HIF-1 signaling pathway [40], Toll-like receptor signaling pathway [41], FoxO signaling pathway [42], focal adhesion [43], and NOD-like receptor signaling pathway [44]. These results were also consistent with what we predicted. In addition, SZF might have potential therapeutic effects on diseases such as cancer, hepatitis, influenza, leishmaniasis, pertussis, and tuberculosis according to the KEGG enrichment analysis. Just as it was reported that different diseases had common or similar pathological changes and could be treated with the same prescription [45], the above results suggested that SZF concentrated more on the systematicness of the body when treating DKD. In other words, SZF possibly regulated the body to reach the balance state, then reaching the aim of treatment.

## 5. Conclusion

In conclusion, this study based on the network pharmacology had preliminarily explained the anti-DKD mechanism of SZF from the perspective of multi-active ingredients, multi-targets, and multi-pathway. In the future, we will further investigate its mechanism by molecular docking, using *in vitro* or *in vivo* studies.

## Figures and Tables

**Table 1:** Common targets of SZF and DKD (50 of 182 targets).

Serial number	Target	Common name	Uniprot ID
1	Aldose reductase	AKR1B1	P15121
2	Acyl coenzyme A:cholesterol acyltransferase	CES1	P23141
3	Signal transducer and activator of transcription 3	STAT3	P40763
4	Protein-tyrosine phosphatase 1C	PTPN6	P29350
5	Vascular endothelial growth factor receptor 2	KDR	P35968
6	Epidermal growth factor receptor erbB1	EGFR	P00533
7	PI3-kinase p110-alpha subunit	PIK3CA	P42336
8	c-Jun N-terminal kinase 1	MAPK8	P45983
9	LXR-alpha	NR1H3	Q13133
10	Estrogen receptor alpha	ESR1	P03372
11	Testis-specific androgen-binding protein	SHBG	P04278
12	Cytochrome P450 2C19	CYP2C19	P33261
13	Protein-tyrosine phosphatase 1B	PTPN1	P18031
14	Butyrylcholinesterase	BCHE	P06276
15	Vitamin D receptor	VDR	P11473
16	Glucose-6-phosphate 1-dehydrogenase	G6PD	P11413
17	Peroxisome proliferator-activated receptor alpha	PPARA	Q07869
18	Peroxisome proliferator-activated receptor delta	PPARD	Q03181
19	Peroxisome proliferator-activated receptor gamma	PPARG	P37231
20	UDP-glucuronosyltransferase 2B7	UGT2B7	P16662
21	11-Beta-hydroxysteroid dehydrogenase 2	HSD11B2	P80365
22	NADPH oxidase 4	NOX4	Q9NPH5
23	Tyrosine-protein kinase SYK	SYK	P43405
24	Glycogen synthase kinase-3 beta	GSK3B	P49841
25	Matrix metalloproteinase 9	MMP9	P14780
26	Matrix metalloproteinase 2	MMP2	P08253
27	Matrix metalloproteinase 12	MMP12	P39900
28	ATP-binding cassette sub-family G member 2	ABCG2	Q9UNQ0
29	P-glycoprotein 1	ABCB1	P08183
30	Arachidonate 12-lipoxygenase	ALOX12	P18054
31	Cyclooxygenase-2	PTGS2	P35354
32	Insulin-like growth factor I receptor	IGF1R	P08069
33	Myeloperoxidase	MPO	P05164
34	Matrix metalloproteinase 3	MMP3	P08254
35	Serine/threonine-protein kinase AKT	AKT1	P31749
36	Beta-secretase 1	BACE1	P56817
37	Tyrosine-protein kinase receptor UFO	AXL	P30530
38	NUAK family SNF1-like kinase 1	NUAK1	O60285
39	Aldehyde reductase	AKR1A1	P14550
40	Plasminogen	PLG	P00747
41	PI3-kinase p110-delta subunit	PIK3CD	O00329
42	PI3-kinase p110-gamma subunit	PIK3CG	P48736
43	Hematopoietic prostaglandin D synthase	HPGDS	O60760
44	Serine-protein kinase ATM	ATM	Q13315
45	Cytochrome P450 24A1	CYP24A1	Q07973
46	Mineralocorticoid receptor	NR3C2	P08235
47	Cannabinoid receptor 1	CNR1	P21554
48	Hepatocyte nuclear factor 4-alpha	HNF4A	P41235
49	C-C chemokine receptor type 1	CCR1	P32246
50	Histone-lysine N-methyltransferase EZH2	EZH2	Q15910

Organism: *Homo sapiens*. Only 50 potential targets' information is shown here, and the whole is in Table 3.

**Table 2:** A total of 182 common targets.

No.	Target	Common name	Uniprot ID
1	Aldose reductase	AKR1B1	P15121
2	Acyl coenzyme A:cholesterol acyltransferase	CES1	P23141
3	Signal transducer and activator of transcription 3	STAT3	P40763
4	Protein-tyrosine phosphatase 1C	PTPN6	P29350
5	Vascular endothelial growth factor receptor 2	KDR	P35968
6	Epidermal growth factor receptor erbB1	EGFR	P00533
7	PI3-kinase p110-alpha subunit	PIK3CA	P42336
8	c-Jun N-terminal kinase 1	MAPK8	P45983
9	LXR-alpha	NR1H3	Q13133
10	Estrogen receptor alpha	ESR1	P03372
11	Testis-specific androgen-binding protein	SHBG	P04278
12	Cytochrome P450 2C19 13	CYP2C19	P33261
13	Protein-tyrosine phosphatase 1B	PTPN1	P18031
14	Butyrylcholinesterase	BCHE	P06276
15	Vitamin D receptor	VDR	P11473
16	Glucose-6-phosphate 1-dehydrogenase	G6PD	P11413
17	Peroxisome proliferator-activated receptor alpha	PPARA	Q07869
18	Peroxisome proliferator-activated receptor delta	PPARD	Q03181
19	Peroxisome proliferator-activated receptor gamma	PPARG	P37231
20	UDP-glucuronosyltransferase 2B7	UGT2B7	P16662
21	11-beta-hydroxysteroid dehydrogenase 2	HSD11B2	P80365
22	NADPH oxidase 4	NOX4	Q9NPH5
23	Tyrosine-protein kinase SYK	SYK	P43405
24	Glycogen synthase kinase-3 beta	GSK3B	P49841
25	Matrix metalloproteinase 9	MMP9	P14780
26	Matrix metalloproteinase 2	MMP2	P08253
27	Matrix metalloproteinase 12	MMP12	P39900
28	ATP-binding cassette sub-family G member 2	ABCG2	Q9UNQ0
29	P-glycoprotein 1	ABCB1	P08183
30	Arachidonate 12-lipoxygenase	ALOX12	P18054
31	Cyclooxygenase-2	PTGS2	P35354
32	Insulin-like growth factor I receptor	IGF1R	P08069
33	Myeloperoxidase	MPO	P05164
34	Matrix metalloproteinase 3	MMP3	P08254
35	Serine/threonine-protein kinase AKT	AKT1	P31749
36	Beta-secretase 1	BACE1	P56817
37	Tyrosine-protein kinase receptor UFO	AXL	P30530
38	NUAK family SNF1-like kinase 1	NUAK1	060285
39	Aldehyde reductase (by homology)	AKR1A1	P14550
40	Plasminogen	PLG	P00747
41	PI3-kinase p110-delta subunit	PIK3CD	O00329
42	PI3-kinase p110-gamma subunit	PIK3CG	P48736
43	Hematopoietic prostaglandin D synthase	HPGDS	O60760
44	Serine-protein kinase ATM	ATM	Q13315
45	Cytochrome P450 24A1	CYP24A1	Q07973
46	Mineralocorticoid receptor	NR3C2	P08235
47	Cannabinoid receptor 1	CNR1	P21554
48	Hepatocyte nuclear factor 4-alpha	HNF4A	P41235
49	C-C chemokine receptor type 1	CCR1	P32246
50	Histone-lysine N-methyltransferase EZH2	EZH2	Q15910
51	MAP kinase p38 alpha	MAPK14	Q16539
52	Bromodomain-containing protein 2	BRD2	P25440
53	Aldehyde dehydrogenase	ALDH2	P05091
54	Fatty acid binding protein adipocyte	FABP4	P15090
55	Fatty acid-binding protein, liver	FABP1	P07148
56	Acyl-CoA desaturase	SCD	O00767
57	MAP kinase ERK1	MAPK3	P27361
58	Short transient receptor potential channel 6	TRPC6	Q9Y210
59	Mitogen-activated protein kinase kinase kinase 5	MAP3K5	Q99683
60	Disintegrin and metalloproteinase domain-containing protein 17	ADAM17	P78536
61	Hexokinase type IV	GCK	P35557
62	Intercellular adhesion molecule-1	ICAM1	P05362
63	P-selectin	SELP	P16109
64	Leukocyte adhesion molecule-1	SELL	P14151
65	Matrix metalloproteinase 1	MMP1	P03956
66	Matrix metalloproteinase 8	MMP8	P22894
67	Endothelin-converting enzyme 1	ECE1	P42892
68	Integrin beta-3	ITGB3	P05106
69	Phosphatidylinositol 4,5-bisphosphate 3-kinase catalytic subunit beta isoform	PIK3CB	P42338
70	Sorbitol dehydrogenase	SORD	Q00796
71	MAP kinase ERK2	MAPK1	P28482
72	Vascular endothelial growth factor receptor 1	FLT1	P17948
73	Matrix metalloproteinase 7	MMP7	P09237
74	Type-1 angiotensin II receptor	AGTR1	P30556
75	Glucose transporter	SLC2A1	P11166
76	Nerve growth factor receptor Trk-A	NTRK1	P04629
77	Tyrosine-protein kinase JAK1	JAK1	P23458
78	Tyrosine-protein kinase JAK2	JAK2	O60674
79	Sodium/glucose cotransporter 2	SLC5A2	P31639
80	Serine/threonine-protein kinase receptor R3	ACVRL1	P37023
81	Epoxide hydratase	EPHX2	P34913
82	Cytochrome P450 11B2	CYP11B2	P19099
83	Endothelin receptor ET-A	EDNRA	P25101
84	Glutathione S-transferase Mu 1	GSTM1	P09488
85	Interleukin-1 beta	ILIB	P01584
86	Insulin receptor	INSR	P06213
87	Protein tyrosine kinase 2 beta	PTK2B	Q14289
88	Cyclooxygenase-1	PTGS1	P23219
89	Cytochrome P450 2C9	CYP2C9	P11712
90	Cytochrome P450 3A4	CYP3A4	P08684
91	Trypsin I	PRSS1	P07477
92	C-C chemokine receptor type 5	CCR5	P51681
93	Dopamine D2 receptor	DRD2	P14416
94	Cholesteryl ester transfer protein	CETP	P11597
95	Calcitonin gene-related peptide type 1 receptor	CALCRL	Q16602
96	Serotonin 2a (5-HT2a) receptor	HTR2A	P28223
97	Disintegrin and metalloproteinase domain-containing protein 10	ADAM10	O14672
98	TGF-beta receptor type I	TGFBR1	P36897
99	Nitric-oxide synthase, brain	NOS1	P29475
100	Cathepsin (B and K)	CTSB	P07858
101	Bradykinin B1 receptor	BDKRB1	P46663
102	Potassium voltage-gated channel subfamily KQT member 1	KCNQ1	P51787
103	Leukotriene A4 hydrolase	LTA4H	P09960
104	Apoptosis regulator Bcl-2	BCL2	P10415
105	Kininogen-1	KNG1	P01042
106	Solute carrier family 22 member 2	SLC22A2	O15244
107	Plasma retinol-binding protein	RBP4	P02753
108	Histone deacetylase 4	HDAC4	P56524
109	Dopamine D3 receptor	DRD3	P35462
110	C-C chemokine receptor type 2	CCR2	P41597
111	Solute carrier family 22 member 12	SLC22A12	Q96S37
112	Glucagon-like peptide 1 receptor	GLP1R	P43220
113	Dual specificity mitogen-activated protein kinase kinase 2	MAP2K2	P36507
114	Death-associated protein kinase 2	DAPK2	Q9UIK4
115	Bile acid receptor FXR	NR1H4	Q96RI1
116	Interleukin-6	IL6	P05231
117	Transcription factor AP-1	JUN	P05412
118	Vascular endothelial growth factor A	VEGFA	P15692
119	Interleukin-10	IL10	P22301
120	Endothelin-1	EDN1	P05305
121	Nitric oxide synthase, endothelial	NOS3	P29474
122	Urotensin II receptor	UTS2R	Q9UKP6
123	78 kDa glucose-regulated protein	HSPA5	P11021
124	Galectin-3	LGALS3	P17931
125	Macrophage migration inhibitory factor	MIF	P14174
126	Serum paraoxonase/arylesterase 1	PON1	P27169
127	Kallikrein 1	KLK1	P06870
128	Rho-associated protein kinase 1	ROCK1	Q13464
129	Sphingosine kinase 1	SPHK1	Q9NYA1
130	Serine/threonine-protein kinase Sgk1	SGK1	O00141
131	Low affinity sodium-glucose cotransporter	SLC5A4	Q9NY91
132	Neutrophil cytosol factor 1	NCF1	P14598
133	Antileukoproteinase	SLPI	P03973
134	Signal transducer and activator of transcription 1-alpha/beta	STAT1	P42224
135	Protein kinase C beta type	PRKCB	P05771
136	Gap junction alpha-1 protein	GJA1	P17302
137	C-X-C motif chemokine 11	CXCL11	O14625
138	Interleukin-8	CXCL8	P10145
139	Superoxide dismutase [Cu-Zn]	SOD1	P00441
140	C-C motif chemokine 2	CCL2	P13500
141	Hypoxia-inducible factor 1-alpha	HIF1A	Q16665
142	Caveolin-1	CAV1	Q03135
143	Interleukin-1 alpha	IL1A	P01583
144	Nuclear factor erythroid 2-related factor 2	NFE2L2	Q16236
145	C-X-C motif chemokine 10	CXCL10	P02778
146	Plasminogen activator inhibitor 1	SERPINE1	P05121
147	Osteopontin	SPP1	P10451
148	Bone morphogenetic protein 2	BMP2	P12643
149	Transforming growth factor beta-1 proprotein	TGFB1	P01137
150	Cyclin-dependent kinase inhibitor 2A	CDKN2A	P42771
151	Transcription factor E2F1	E2F1	Q01094
152	Thrombomodulin	THBD	P07204
153	Insulin-like growth factor II	IGF2	P01344
154	Catalase	CAT	P04040
155	Phosphatidylinositol 3,4,5-trisphosphate 3-phosphatase and dual-specificity protein phosphatase PTEN	PTEN	P60484
156	Pro-epidermal growth factor	EGF	P01133
157	ATP synthase subunit beta, mitochondria	ATP5F1B	P06576
158	NAD-dependent protein deacetylase sirtuin-1	SIRT1	Q96EB6
159	Angiotensin-converting enzyme	ACE	P12821
160	Matrix metalloproteinase 10	MMP10	P09238
161	Transketolase	TKT	P29401
162	Dipeptidyl peptidase IV	DPP4	P27487
163	Nuclear factor NF-kappa-B p65 subunit	RELA	Q04206
164	Nitric oxide synthase, inducible	NOS2	P35228
165	Protein kinase C alpha type	PRKCA	P17252
166	Tumor necrosis factor	TNF	P01375
167	Protein kinase C epsilon type	PRKCE	Q02156
168	Renin	REN	P00797
169	Axin1/beta-catenin	CTNNB1	P35222
170	Fibronectin	FN1	P02751
171	C-X-C chemokine receptor type 4	CXCR4	P61073
172	Heparanase	HPSE	Q9Y251
173	Glucagon	GCG	P01275
174	Tumor necrosis factor receptor superfamily member 11B	TNFRSF11B	O00300
175	Metalloproteinase inhibitor 1	TIMP1	P01033
176	Metalloproteinase inhibitor 2	TIMP2	P16035
177	Fibroblast growth factor 2	FGF2	P09038
178	Lipoprotein lipase	LPL	P06858
179	Coagulation factor V	F5	P12259
180	Cyclic AMP-responsive element-binding protein 1	CREB1	P16220
181	Phosphatidylinositol 3,4,5-trisphosphate 5- phosphatase 2	INPPL1	O15357
182	Tumor necrosis factor ligand superfamily member 6	FASLG	P48023

**Table 3:** Thirty key targets obtained by network topology analysis.

Serial number	Node	Degree	Closeness centrality	Betweenness centrality
1	PIK3CA	40	0.49508197	0.09370214
2	STAT3	40	0.5	0.0863086
3	AKT1	35	0.49025974	0.15311921
4	KNG1	33	0.44023324	0.06128185
5	VEGFA	33	0.49185668	0.06953442
6	JUN	32	0.48089172	0.07229449
7	MAPK3	30	0.4617737	0.02240476
8	MAPK1	30	0.4689441	0.06714477
9	EGF	27	0.4617737	0.0336672
10	EDN1	27	0.46604938	0.05180077
11	EGFR	26	0.44023324	0.01794429
12	JAK1	26	0.44940476	0.02254905
13	IL6	26	0.45209581	0.02532622
14	CXCL8	25	0.43768116	0.03191743
15	RELA	24	0.45757576	0.04035241
16	FN1	23	0.4351585	0.01464828
17	JAK2	23	0.44940476	0.01620852
18	CTNNB1	23	0.45481928	0.06488997
19	TNF	22	0.44281525	0.0272631
20	TGFB1	21	0.44281525	0.03270724
21	MMP9	20	0.40921409	0.03200512
22	CXCR4	19	0.41032609	0.01402652
23	TIMP1	19	0.41712707	0.00798146
24	MAPK14	19	0.44411765	0.01628416
25	BDKRB1	18	0.3994709	0.00725308
26	PIK3CB	18	0.40921409	0.00732155
27	MAPK8	18	0.42296919	0.03099697
28	ITGB3	18	0.42296919	0.01050834
29	CCR5	16	0.39841689	0.00592392
30	PLG	16	0.40266667	0.02168017

**Table 4:** The information of active ingredients.

No.	Active ingredients	Code name
1	Isoimperatorin	M1
2	1,2,5,6-Tetrahydrotanshinone	M2
3	5,6-Dihydroxy-7-isopropyl-1,1-dimethyl-2,3-dihydrophenanthren-4-one	M3
4	(E)-3-[2-(3,4-Dihydroxyphenyl)-7-hydroxy-benzofuran-4-yl]acrylic	M4
5	2-(4-Hydroxy-3-methoxyphenyl)-5-(3-hydroxypropyl)-7-methoxy-3-benzofurancarboxaldehyde	M5
6	Przewaquinone c	M6
7	Cryptotanshinone	M7
8	Dihydrotanshinlactone	M8
9	Isotanshinone II	M9
10	Miltipolone	M10
11	Miltirone	M11
12	Tanshinaldehyde	M12
13	Danshenol B	M13
14	Danshenol A	M14
15	Deoxyneocryptotanshinone	M15
16	Dihydrotanshinone I	M16
17	Miltionone I	M17
18	Miltionone II	M18
19	Neocryptotanshinone ii	M19
20	Neocryptotanshinone	M20
21	Luteolin	M21
22	Salvilenone I	M22
23	Salviolone	M23
24	Epidanshenspiroketallactone	M24
25	Tanshinone iia	M25
26	*α*-Amyrin	M26
27	Dan-shexinkum d	M27
28	Sclareol	M28
29	Dehydrotanshinone II A	M29
30	Baicalin	M30
31	2-Isopropyl-8-methylphenanthrene-3,4-dione	M31
32	Formyltanshinone	M32
33	3-Beta-Hydroxymethyllenetanshiquinone	M33
34	Methylenetanshinquinone	M34
35	(2R)-3-(3,4-Dihydroxyphenyl)-2-[(Z)-3-(3,4-dihydroxyphenyl)acryloyl]oxy-propionic acid	M35
36	(6S)-6-(Hydroxymethyl)-1,6-dimethyl-8,9-dihydro-7H-naphtho[8,7-g]benzofuran-10,11-dione	M36
37	Tanshinone VI	M37
38	Przewalskin b	M38
39	6-o-Syringyl-8-o-acetyl shanzhiside methyl ester	M39
40	Prolithospermic acid	M40
41	(Z)-3-[2-[(E)-2-(3,4-Dihydroxyphenyl)vinyl]-3,4-dihydroxyphenyl]acrylic acid	M41
42	Salvianolic acid g	M42
43	Salvianolic acid j	M43
44	Danshenspiroketallactone	M44
45	1-Methyl-8,9-dihydro-7H-naphtho[5,6-g]benzofuran-6,10,11-trione	M45
46	3,9-di-O-MethylnissolinM	M46
47	(6aR,11aR)-9,10-Dimethoxy-6a,11a-dihydro-6H-benzofurano[3,2-c]chromen-3-ol	M47
48	(3R)-3-(2-Hydroxy-3,4-dimethoxyphenyl)chroman-7-ol	M48
49	Isorhamnetin	M49
50	Kaempferol	M50
51	Quercetin	M51
52	Jaranol	M52
53	Bifendate	M53
54	Formononetin	M54
55	Isoflavanone	M55
56	Calycosin	M56
57	Hederagenin	M57
58	Sennoside E_qt	M58
59	Toralactone	M59
60	Palmidin A	M60
61	Daucosterol_qt	M61
62	Eupatin	M62
63	Procyanidin B-5,3'-O-gallate	M63
64	Rhein	M64
65	Beta-sitosterol	M65
66	Aloe-emodin	M66
67	Lipase	M67
68	Gardnerilin a	M68
69	Hirudin	M69
70	o-Desulfated heparin	M70
71	Ursolic acid	M71
72	Heparin	M72
73	Genioisidic acid	M73
74	Genipinic acid	M74
75	Nadroparin	M75

## Data Availability

The data used to support the results of this study can be obtained from the corresponding author upon reasonable request.
